# Neonatal and infant mortality after maternal influenza and pertussis vaccination: Probabilistically linked cohort study

**DOI:** 10.1080/21645515.2025.2587307

**Published:** 2025-11-20

**Authors:** Mohinder Sarna, Christopher C. Blyth, Hannah C. Moore, Gavin Pereira, Lisa McHugh, Michael Binks, Karin Lust, Paul Van Buynder, Damien Foo, Ross Andrews, Annette K. Regan

**Affiliations:** a Wesfarmers Centre for Vaccines and Infectious Diseases, The Kids Research Institute Australia, University of Western Australia, Nedlands, Australia; b Curtin School of Population Health, Curtin University, Bentley, Australia; c Department of Paediatric Infectious Diseases, Perth Children’s Hospital, School of Medicine, University of Western Australia, Nedlands, Australia; d Department of Microbiology, Pathwest Laboratory Medicine, QEII Medical Centre, Nedlands, Australia; e enAble Institute, Curtin University, Bentley, Australia; f School of Public Health, University of Queensland, Brisbane, Australia; g Child and Maternal Health Division, Menzies School of Health Research, Charles Darwin University, Casuarina, Australia; h Women and Kids Theme, South Australian Health and Medical Research Institute, Adelaide, South Australia, Australia; i Department of Obstetric Medicine, Royal Brisbane and Women’s Hospital, Brisbane, Australia; j School of Medicine, University of Queensland, Brisbane, Australia; k School of Medicine, Griffith University, Southport, Australia; l Curtin Health Nexus, Faculty of Health Sciences, Curtin University, Bentley, Australia; m Yale School of the Environment, Yale University, New Haven, CT, USA; n Communicable Disease Control Branch, Queensland Health, Brisbane, Australia; o Department of Research & Evaluation, Kaiser Permanente Southern California, Pasadena, CA, USA; p Fielding School of Public Health, University of California Los Angeles, Los Angeles, CA, USA

**Keywords:** Maternal vaccines, influenza, pertussis, vaccine safety, mortality

## Abstract

Maternal influenza and pertussis vaccination is an important strategy to reduce morbidity and mortality in infants. Previous vaccine safety studies have mostly focused on the association between maternal vaccination and fetal death. We conducted a probabilistically linked cohort study of mother-infant pairs in three Australian jurisdictions: Queensland, Northern Territory, and Western Australia, to evaluate the risk of all-cause neonatal and infant mortality associated with seasonal influenza and pertussis maternal vaccination. Vaccination records were obtained from regional immunization databases, and mortality information was obtained from perinatal databases and death registries from 2015 to 2017. Cox proportional hazard models with vaccination as time-varying exposure and weighted by the inverse probability of treatment were used to estimate adjusted hazard ratios (aHR). Among 252,924 mothers and 277,979 liveborn infants, 8,288 (3.0%) infants were exposed *in utero* to influenza vaccine only, 88,011 (31.7%) to pertussis vaccine only, and 67,036 (24.1%) to both vaccines. There were 741 all-cause infant deaths (crude mortality rate 2.67 deaths/1,000 live births). Influenza and pertussis vaccines were associated with a reduction in infant mortality when given alone (aHR [influenza]: 0.66, 95%CI 0.43, 0.99 and aHR [pertussis]: 0.69, 95%CI 0.54, 0.87), or in combination (aHR: 0.58; 95%CI 0.44, 0.77), predominantly attributed to reduction in early neonatal (0–7 d) death. No association was observed between influenza or pertussis vaccine and post-neonatal (8–28 d) mortality. We observed insufficient evidence of greater neonatal or infant mortality associated with maternal influenza or pertussis vaccination. These findings could be useful to healthcare professionals when counseling pregnant patients on maternal vaccination.

## Introduction

Neonates and infants under 6 months of age have immature immune systems and are highly susceptible to influenza and pertussis infections.^1,2^ They are not adequately protected from these infections until they have completed their primary vaccination series, which leaves a period of vulnerability. Influenza vaccines are currently not licensed for infants <6 months of age, and at least two doses of infant pertussis vaccination are required for protection against severe pertussis (at 2 and 4 months). Maternal influenza and pertussis vaccines increase the level of transplacentally transferred maternal antibodies to the newborn and extend the duration of protection through passive immunity and are therefore an important strategy for reducing morbidity and mortality in neonates.^3,4^

In Australia, inactivated influenza vaccines have been recommended during any stage of pregnancy since 2000 and funded for pregnant women since 2010.^5^ The Australian Influenza Vaccine Committee (AIVC) provides advice to the Therapeutic Goods Administration on the composition of the seasonal influenza vaccine to be supplied each year.^6^ The AIVC adopts the World Health Organization’s recommendations each year. Strain composition is national, and States and Territories use these same strains. Between 2014 and 2015, States and Territories in Australia introduced jurisdictionally funded maternal trivalent pertussis vaccination programs, to be administered at approximately 28 weeks gestation.^7^ Pertussis vaccines licensed and available for States and Territories to use at a jurisdictional level are determined by the Australian Technical Advisory Group on Immunisation, a technical advisory group of the Australian Government that advises the Minister of Health and Aged Care. While jurisdictions may support state-based programs, they are limited to vaccines licensed and approved for use by ATAGI. Maternal pertussis vaccines were subsequently federally funded and incorporated into the National Immunization Program (NIP) in July 2018.^5,7^ Uptake of influenza vaccines in Australia was low in the years prior to the introduction of maternal pertussis vaccines and has improved following the introduction of maternal pertussis vaccination programs. Between 2016 and 2021, coverage across Australian jurisdictions ranged between 58 and 90%,^8^ with immunization rates among First Nations (Aboriginal and/or Torres Strait Islander) women lagging behind those among non-Aboriginal women.^8,9^

Previous vaccine safety studies have examined the association between maternal influenza vaccination and fetal death before and after 20 weeks, with most studies reporting no association between fetal death or preterm birth and seasonal or pandemic maternal influenza vaccination.^1,10,11^ Relatively few studies have examined any association between maternal vaccination and neonatal (0–28 d) or infant mortality.^12,13^ Furthermore, few studies have examined the safety of seasonal influenza vaccines and pertussis vaccines when administered in the same pregnancy.^13,14^ Confirming that vaccines do not increase neonatal or infant death risk is critical for maintaining public confidence in maternal immunization programs. Furthermore, as neonatal and infant mortality are rare outcomes in high-income countries, large, well-designed studies are needed to detect rare adverse events. Outcomes such as reduced neonatal all-cause mortality can also quantify the life benefits of vaccination. In low to middle income countries where neonatal mortality is higher and maternal vaccination coverage is lower, demonstrating beneficial effects of maternal vaccination on all-cause mortality can target high-risk populations and reduce infant deaths globally.

The aim of this study was to compare the risk of all-cause neonatal and infant mortality among offspring of mothers who received an influenza and/or a pertussis vaccine during pregnancy with offspring of unvaccinated mothers.

## Methods

### Study design, setting, and cohort description

We conducted an observational, population-based cohort study of resident mother-infant pairs using probabilistically and deterministically linked data collected in administrative health databases from three jurisdictions in Australia: Queensland (Qld), Northern Territory (NT), and Western Australia (WA).^15^ Together these three jurisdictions accounted for 33% of Australia’s annual birth cohort.^16^ The population profile and estimated vaccine coverage of both vaccines in these jurisdictions have been outlined previously.^15^ Births recorded between 1 January 2015 to 31 December 2017 were included in the cohort, a period of time where both influenza and pertussis maternal vaccination programs had been introduced, recommended and fully funded (free) under the NIP. Influenza virus circulation in the study years peaked in the winter months of June–August, with less distinct and atypical seasonality in northern regions of the country.^17^ In Australia, seasonal influenza vaccines are typically available beginning in April each year. Pertussis epidemiology in Australia exhibits seasonal variation, although pertussis activity can occur year-round. Cases generally increase late spring through summer (November to January).^18^ Australia also has cyclical epidemics of pertussis every 3–5 y. Pertussis activity in Northern Australia exhibits the same seasonal pattern, although somewhat blunted seasonality compared to more temperate jurisdictions.

### Data sources

Singleton, liveborn infants were identified in jurisdictional birth registers and perinatal data collections (Supplementary Table S1). Mothers and infants were probabilistically linked to jurisdictional vaccination data and mortality registers. Perinatal data included maternal demographics and health information, obstetric history, date of delivery, gestational age, and birthweight for all infants born at least 20 weeks’ gestation or birthweight of ≥400 g (where gestational age is unknown). Death registrations included the date of all registered deaths. The final underlying cause of death was provided by the Australian Bureau of Statistics and coded using the World Health Organizations’ International Statistical Classification of Diseases and Related Health Problems, 10^th^ edition (ICD-10) diagnostic codes (Supplementary Table S2). Only the principal cause of death was considered when presenting the proportion of infants who died by specific causes.

Individual-level data on maternal vaccinations were obtained from immunization registers (QLD: 2015–2017, NT: 2015–2017, WA: 2015–2016) and jurisdictional perinatal data collections (QLD: 2015–2017, WA: 2016–2017). Data from immunization registers included the date of vaccination, vaccine brand, and batch number. Data from perinatal data collections included the estimated gestation at which vaccinations were administered as reported by their healthcare provider. These data collections were used to inform the trimester of administration of vaccination. All singleton, liveborn children born between 1 January 2015 and 31 December 2017 were included, with the exception of WA, where data were available to 31 August 2017. Linkage was carried out by the Data Services section of Queensland Health for Qld data, by the SANT Datalink for NT data, and Data Services of the Western Australian Department of Health for WA data.

### Vaccine composition

*Influenza*: while the AIVC adopts influenza vaccine strain composition recommendations made by the World Health Organization each year (Supplementary Table S3), and vaccine availability is national, our analysis of the data on vaccine brands administered show that jurisdictions administered both trivalent and quadrivalent influenza vaccines during the study period.

*Pertussis*: Both Boostrix® (GSK) and Adacel® (Sanofi Pasteur) were available for use for adults/adolescents in Australia during the study period. All jurisdictions used both vaccines. Both formulations contain pertussis toxoid, filamentous hemagglutinin, and pertactin pertussis antigens. Adacel® also includes fimbriae types 2 and 3. There were also two mothers in the NT who received combination vaccines (Adacel®-IPV and Boostrix®-IPV). Vaccine brand information on maternal influenza and pertussis vaccines recorded in perinatal data collections in the NT and QLD was not available.

### Ethics

The study received approval and a waiver of consent from the Royal Brisbane and Women’s Hospital Human Research Ethics Committee in Qld (HREC/2018/QRBW/47660), Menzies School of Health Research in the NT (HREC 2018-3199), the WA Department of Health Human Research Ethics Committee (RA#2016.56), and the Curtin University Human Research Ethics Committee (RA#2017-0808) in WA. Ethics approval was also sought from the Western Australian Aboriginal Health Ethics Committee (#889). The study uses the REporting of studies Conducted using Observational Routinely collected Data (RECORD) guideline.^19^

### Exposure measurement

Receipt of maternal influenza and/or pertussis vaccination anytime during pregnancy was the exposure of interest. Pregnancy was defined as the period between the estimated date of conception (calculated from the best clinical estimate of gestational age derived by ultrasound and subtracted from the gestational age) and the infant’s date of birth. Women with no vaccination record, those with a vaccination date before the date of conception or after the date of birth, and women vaccinated less than 14 d before birth were considered ‘unvaccinated.’

### Outcomes

Our primary outcome measure was death due to any cause during the first 365 d of life (i.e., infant mortality). We separately assessed early neonatal death (death occurring 0–7 d after birth), late neonatal death (death occurring 8–28 d after birth), and post-neonatal death (death occurring 29–365 d after birth). Specific causes of death were identified from mortality registers classified using ICD-10 diagnostic codes (Supplementary Table S2).

### Covariates

Maternal covariates included maternal age at delivery, First Nations status, socioeconomic status, parity, preexisting medical conditions (e.g., preexisting diabetes, essential hypertension, and asthma), smoking during pregnancy, pregnancy complications (gestational diabetes, gestational hypertension, and pre-eclampsia), and gestational age at the first antenatal care visit. In Qld and the NT, Aboriginal status was validated and recorded in perinatal data collections. In WA, Aboriginal status was defined using the validated ‘Getting Our Stories Right’ algorithm, drawing from multiple administrative datasets, provided by the Data Services at the WA Department of Health.^20^

Socioeconomic status was defined using the Socioeconomic Index for Areas (SEIFA) measure of relative socioeconomic advantage and disadvantage, which is an area-based measure of relative access to resources for households within the same census collection district.^21^ SEIFA was divided into deciles, with decile 1 being the most disadvantaged and decile 10 being the most advantaged. Records with missing information on covariates were excluded from the analysis. All covariates were selected *a priori* based on their association with maternal vaccination.^1,13^

### Exclusions

We excluded plural births, stillbirths, infants with birthweight <400 g or missing birthweight, and infants with missing gestational age or gestational age <20 weeks.

### Statistical analysis

Inverse probability of treatment weights were applied to Cox proportional hazard models to estimate the unadjusted and adjusted hazard ratios (aHRs) and 95% confidence intervals comparing the rate of death between infants of vaccinated and unvaccinated mothers. We estimated the predicted probability of treatment (vaccination) using multivariable logistic regression with maternal covariates as predictors to construct the inverse probability treatment weights. To preserve temporality in our multivariable model, time-varying predictors, like pregnancy complications, were considered as a predictor only when they could have occurred before vaccination and therefore could have influenced the probability of vaccination. For example, if vaccination occurred in the first trimester and pre-eclampsia was diagnosed (predominantly at 24 weeks) we did not consider preeclampsia predictive of vaccination. Inverse probability weights that exceeded the first and 99^th^ percentiles were trimmed. We confirmed balance in baseline characteristics between vaccinated and unvaccinated subjects after weighting by calculating the standardized mean differences of each covariate. A difference of <10% was considered ‘balanced’ (Supplementary Figure S1).^22^ Robust standard errors were calculated to adjust for more than one birth per mother.

The underlying timescale was infant age in days. Time-at-risk commenced at birth and ended at the earliest of a) 12 months of age, b) the last date of available data (end of the study period), c) the date of death. Adjusted models additionally controlled for the child’s gestational age at birth. The main analysis included all liveborn, singleton births. In sensitivity analyses, we separately analyzed term (≥37 weeks) and preterm (<37 weeks) infants, as prematurity is linked to the likelihood of exposure and outcome. We also conducted further sensitivity analyses omitting deaths occurring on the day of birth to account for potentially misclassified stillbirths.

### Participant involvement

No participants were involved in setting the research question or the exposure or outcome measures nor were they involved in the design and implementation of the study. The study had input from the Kulunga Aboriginal Unit at The Kids Research Institute, and the Healthy Pregnancies community reference groups at Curtin University.

## Results

**Figure 1.**
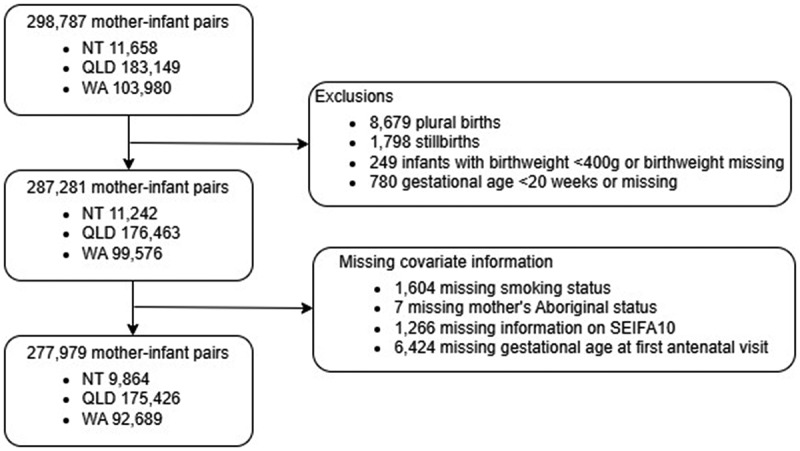
Flow diagram of exclusions and final study cohort.

A total of 298,787 births were identified from the three jurisdictions with a date of birth between 1 January 2015 to 31 December 2017 (Figure 1). Of these, 8,679 plural births, 1,798 stillbirths, 249 infants with birthweight <400 g or missing birthweight, and 780 infants with missing gestational age or gestational age <20 weeks were excluded (Figure 1). An additional 9,302 (3.1%) infants were excluded if covariate information was missing. The final cohort comprised 277,979 liveborn singletons from 252,924 mothers, 257,402 born at term and 20,577 (7.4%) infants born preterm. Maternal characteristics between vaccinated and unvaccinated mothers were balanced following inverse probability of treatment weighting (Supplementary Figure S1).

Overall, 8,288 (3.0%) infants were exposed *in utero* to influenza vaccine only, 88,011 (31.7%) to pertussis vaccine only, and 67,036 (24.1%) to both influenza and pertussis vaccine (Table 1). Most vaccinated mothers received influenza and pertussis vaccines during the third trimester (n = 37,692, 50.1% and n = 138,180, 89.3% respectively). Vaccination was positively associated with earlier initiation of prenatal care, Aboriginal status, primiparity and greater socioeconomic advantage. Maternal vaccination was negatively associated with prematurity.

**Table 1.** Number and percentage of women vaccinated for influenza and/or pertussis vaccine by maternal demographic characteristics of women in the Links2HealthierBubs cohort, 1 January 2015 – 31 December 2017.

Characteristic	Total (N=277,979) n, (%)	Unvaccinated (N=114,644) n, (%)	Vaccinated		
			Influenza vaccine only (N=8,288) n, (%)	Pertussis vaccine only (N=88,011) n, (%)	Both influenza and pertussis vaccines (N=67,036) n, (%)
**Maternal age at delivery (years)**					
<20 y	9,222 (3.3)	4,488 (3.9)	342 (4.1)	2,617 (3.0)	1,775 (2.6)
20–24 y	40,113 (14.4)	17,708 (15.4)	1,150 (13.9)	12,937 (14.7)	8,318 (12.4)
25–29 y	78,871 (28.4)	31,818 (27.8)	2,233 (26.9)	25,807 (29.3)	19,013 (28.4)
30–34 y	93,484 (33.6)	36,942 (32.2)	2,810 (33.9)	29,595 (33.6)	24,137 (36.0)
≥35 y	56,289 (20.2)	23,688 (20.7)	1,753 (21.2)	17,055 (19.4)	13,793 (20.6)
**Maternal Aboriginal status**					
Aboriginal	19,635 (7.1)	10,676 (9.3)	960 (11.6)	4,690 (5.3)	3,309 (4.9)
Non-Aboriginal	258,344 (92.9)	103,968 (90.7)	7,328 (88.4)	83,321 (94.7)	63,727 (95.1)
**Parity**					
Primiparous	115,287 (41.5)	39,638 (34.6)	3,145 (37.9)	40,004 (45.5)	32,500 (48.5)
One prior birth	96,012 (34.5)	41,474 (36.2)	3,134 (37.8)	28,976 (32.9)	22,428 (33.5)
>1 prior birth	66,680 (24.0)	33,532 (29.2)	2,009 (24.2)	19,031 (21.6)	12,108 (17.9)
**Smoked during pregnancy**					
No	246,473 (88.7)	97,967 (85.5)	7,274 (87.8)	79,487 (90.3)	61,745 (92.1)
Yes	31,506 (11.3)	16,677 (14.5)	1,014 (12.2)	8,524 (9.7)	5,291 (7.9)
**Season of birth**					
Summer (Dec – Feb)	67,226 (24.2)	32,220 (28.1)	2,139 (25.8)	21,699 (24.7)	11,168 (16.7)
Autumn/Fall (Mar – May)	72,134 (25.9)	35,779 (31.2)	1,216 (14.7)	26,884 (30.5)	8,255 (12.3)
Winter (Jun – Aug)	70,525 (25.4)	25,370 (22.1)	1,986 (24.0)	19,382 (22.0)	23,787 (35.5)
Spring (Sep – Nov)	68,094 (24.5)	21,275 (18.6)	2,947 (35.6)	20,046 (22.8)	23,826 (35.5)
**Infant birth year**					
2015	93,410 (33.6)	59,144 (51.5)	3,125 (37.7)	19,757 (22.4)	11,384 (17.0)
2016	94,075 (33.8)	31,911 (27.8)	2,467 (29.8)	34,835 (39.6)	24,862 (37.1)
2017	90,494 (32.6)	23,589 (20.6)	2,696 (32.5)	33,419 (38.0)	30,790 (45.9)
**Mother’s medical conditions**					
Essential hypertension	4,744 (1.7)	1,876 (1.6)	194 (2.3)	1,344 (1.5)	1,330 (2.0)
Pre-existing diabetes mellitus	2,592 (0.9)	1,134 (1.0)	108 (1.3)	698 (0.8)	652 (1.0)
Asthma	15,505 (5.6)	7,148 (6.2)	552 (6.7)	4,276 (4.9)	3,529 (5.3)
Cardiac conditions	4,448 (1.6)	1,902 (1.7)	176 (2.1)	1,399 (1.6)	971 (1.4)
Renal conditions	939 (0.3)	389 (0.3)	37 (0.4)	296 (0.3)	217 (0.3)
Anaemia	10,928 (3.9)	4,223 (3.7)	402 (4.9)	3,767 (4.3)	2,536 (3.8)
**Pregnancy complications**					
Gestational diabetes	25,741 (9.3)	11,001 (9.6)	930 (11.2)	7,714 (8.8)	6,096 (9.1)
Gestational hypertension	9,331 (3.4)	3,813 (3.3)	287 (3.5)	2,884 (3.3)	2,347 (3.5)
Pre-eclampsia	6,685 (2.4)	2,962 (2.6)	234 (2.8)	1,909 (2.2)	1,580 (2.4)
**First antenatal care visit^a^**					
First trimester	203,618 (73.2)	78,434 (68.4)	6,086 (73.4)	66,772 (75.9)	52,326 (78.1)
Second trimester	67,405 (24.2)	31,717 (27.7)	2,034 (24.5)	19,903 (22.6)	13,751 (20.5)
Third trimester	6,847 (2.5)	4,387 (3.8)	166 (2.0)	1,336 (1.5)	958 (1.4)
**SEIFA ranking^b^**					
Quintile 1 (most disadvantaged)	57,743 (20.8)	27,294 (23.8)	2,005 (24.2)	16,594 (18.9)	11,850 (17.7)
Quintile 2	52,393 (18.8)	22,155 (19.3)	1,446 (17.4)	16,937 (19.2)	11,855 (17.7)
Quintile 3	55,368 (19.9)	22,275 (19.4)	1,522 (18.4)	18,120 (20.6)	13,451 (20.1)
Quintile 4	59,153 (21.3)	22,607 (19.7)	1,710 (20.6)	19,862 (22.6)	14,974 (22.3)
Quintile 5 (least disadvantaged)	53,322 (19.2)	20,313 (17.7)	1,605 (19.4)	16,498 (18.7)	14,906 (22.2)
**Jurisdiction**					
Queensland	175,426 (63.1)	60,162 (52.5)	3,723 (44.9)	66,714 (75.8)	44,827 (66.9)
Northern Territory	9,864 (3.5)	6,677 (5.8)	744 (9.0)	1,708 (1.9)	735 (1.1)
Western Australia	92,689 (33.3)	47,805 (41.7)	3,821 (46.1)	19,589 (22.3)	21,474 (32.0)
**Child characteristics**					
Indigenous^c^	22,707 (8.2)	12,004 (10.5)	1,043 (12.6)	5,747 (6.5)	3,913 (5.8)
Non-Indigenous	254,952 (91.7)	102,408 (89.3)	7,244 (87.4)	82,186 (93.4)	63,114 (94.1)
Term (≥37 weeks)	257,402 (92.6)	103,540 (90.3)	7,405 (89.3)	82,940 (94.2)	63,517 (94.8)
Preterm (<37 weeks)	20,577 (7.4)	11,104 (9.7)	883 (10.7)	5,071 (5.8)	3,519 (5.2)
**Deaths (n, per 1,000)**					
0–365 d post birth	741 (2.7)	536 (4.7)	28 (3.4)	105 (1.2)	72 (1.1)
0–7 d post birth	388 (1.4)	320 (2.8)	13 (1.6)	35 (0.4)	20 (0.3)
8–28 d post birth	86 (0.8)	56 (0.5)	5 (0.6)	17 (0.2)	8 (0.1)
29–365 d post birth	267 (2.4)	160 (1.4)	10 (0.8)	53 (0.6)	44 (0.7)

^a^109 women had no antenatal care; ^b^Socioeconomic Index for Areas in Australia- a ranking based on socioeconomic advantage and disadvantage based on census data^21^; ^c^child Indigenous status missing for 320 children.

There were 741 infant deaths from all causes during the study period (crude mortality rate 2.67 deaths/1,000 live births): 388 early (0–7 d) neonatal deaths (52.4%), 86 late neonatal (8–28 d) deaths (11.6%), and 267 (36.1%) post-neonatal (29–365 d) deaths. The overall median age at death was 6 days (interquartile range [IQR]: 0–71 d). Cause of death was recorded for 528 (71.4%) infants. The principal causes of death were from conditions originating in the perinatal period (n = 227, 40.5%), congenital abnormalities (n = 158, 28.2%), and infections (n = 22, 3.9%). Principal cause of death was missing for 29% of infants. Cause of death information is provided by the National Coronial Information System in by the Victorian Department of Justice and Community Safety and often lags behind death registration data.

Infant mortality occurred in 4.7% of infants born to unvaccinated mothers, 3.4% born to influenza-only vaccinated mothers, 1.2% born to pertussis-only vaccinated mothers, and 1.1% of infants born to mothers vaccinated against influenza and pertussis. The median age at death was 37 d (IQR: 3, 118 d) for infants of vaccinated mothers and 2 d (IQR: 0, 46.5) for infants of unvaccinated mothers. When given alone, influenza and pertussis vaccination was associated with a reduction in the rate of infant mortality (0–365 d) ([aHR]: 0.66; 95%CI 0.43, 0.99 and aHR: 0.69; 95%CI 0.54, 0.87, respectively, Table 2). This difference was predominantly attributed to a reduction in early neonatal death (death 0–7 d after birth) (aHR influenza: 0.46; 95%CI 0.25, 0.83 and aHR pertussis 0.56; 95%CI 0.37, 0.86). We observed no association between receipt of pertussis or influenza vaccination in pregnancy and late neonatal (8–28 d) mortality or post-neonatal mortality (29–365 days) (aHR: 1.17; 95%CI 0.45, 3.07 and aHR: 0.90; 95%CI 0.45, 1.79, respectively, Table 2). We observed similar results when considering influenza and pertussis vaccines given concomitantly (aHR: 0.49; 95%CI 0.27, 0.89 for infants aged 0–7 d, aHR: 0.83; 95%CI 0.54, 1.27 for infants aged 29–365 d).

**Table 2.** Risk of neonatal and infant mortality associated with prenatal exposure to seasonal inactivated influenza vaccine and/or pertussis vaccine by infant age, 1 January 2015 – December 2017.

Age categories	Unexposed infants (N=114,644)	Exposed infants by vaccine type			
		Influenza vaccine only (N=8,288)	Pertussis vaccine only (N=88,011)	Influenza and pertussis vaccine (N=67,036)	Influenza or pertussis vaccine (N=163,335)
***Infant mortality (infants aged 0–365 d)***					
N	114,644	8,288	88,011	67,036	163,335
Deaths, n (no. per 1,000)	536 (4.7)	28 (3.4)	105 (1.2)	72 (1.1)	205 (1.3)
Unweighted HR^a^ (95% CI)	1 [Reference]	0.78 (0.52,1.16)	0.26 (0.21, 0.33)	0.22 (0.17, 0.29)	0.27 (0.23, 0.32)
Weighted aHR (95% CI)^b^	1 [Reference]	0.66 (0.43, 0.99)	0.69 (0.54, 0.87)	0.58 (0.44, 0.77)	0.64 (0.53, 0.76)
***Early neonatal mortality (infants aged 0–7 d)***					
N	114,644	8,288	88,011	67,036	163,335
Deaths, n (no. per 1,000)	320 (2.8)	13 (1.6)	35 (0.4)	20 (0.3)	68 (0.4)
Unweighted HR (95% CI)	1 [Reference]	0.50 (0.27, 0.94)	0.14 (0.10, 0.20)	0.10 (0.06, 0.16)	0.14 (0.11, 0.18)
Weighted aHR (95% CI)^b^	1 [Reference]	0.46 (0.25, 0.83)	0.56 (0.37, 0.86)	0.49 (0.27, 0.89)	0.48 (0.34, 0.67)
***Late neonatal mortality (infants aged 8–28 d)***					
N	113,915	8,195	87,466	66,370	163,267
Deaths, n (no. per 1,000)	56 (0.5)	5 (0.6)	17 (0.2)	8 (0.1)	30 (0.2)
Unweighted HR (95% CI)	1 [Reference]	1.45 (0.58, 3.63)	0.42 (0.24, 0.73)	0.23 (0.10, 0.50)	0.39 (0.25, 0.62)
Weighted aHR (95% CI)^b^	1 [Reference]	1.17 (0.45, 3.07)	0.90 (0.45, 1.79)	0.37 (0.16, 0.87)	0.77 (0.44, 1.35)
***Post-neonatal mortality (infants aged 29–365 d)***					
N	112,703	7,991	85,807	64,324	163,237
Deaths, n (no. per 1,000)	160 (1.4)	10 (0.8)	53 (0.6)	44 (0.7)	107 (0.6)
Unweighted HR (95% CI)	1 [Reference]	1.16 (0.61, 2.20)	0.47 (0.35, 0.65)	0.47 (0.33, 0.68)	0.51 (0.40, 0.66)
Weighted aHR (95% CI)^b^	1 [Reference]	1.04 (0.55, 1.97)	0.75 (0.53, 1.07)	0.83 (0.54, 1.27)	0.81 (0.61, 1.08)

Abbreviations: aHR, adjusted hazard ratio; CI, confidence interval; HR, unadjusted hazard ratio.

^a^Hazard ratios weighted by inverse probability of treatment factoring for maternal covariates including age, Indigenous status, parity, pre-existing medical conditions (asthma, essential hypertension, pre-existing diabetes), pregnancy complications (gestational diabetes, gestational hypertension, pre-eclampsia), smoking status during pregnancy, gestational age at first prenatal care visit, year and season of birth, and socioeconomic advantage as measured by SEIFA quintile^21^; ^b^models were additionally adjusted for the infant’s gestational age.

Similar results were observed for pertussis vaccine given alone (aHR: 0.62; 95%CI 0.46,0.84) or in combination with influenza vaccine among term infants (aHR: 0.65; 95%CI 0.46, 0.93). Results were mixed for preterm infants (pertussis alone: aHR: 0.88; 95%CI 0.57, 1.36 and influenza and pertussis: aHR: 0.75; 95%CI 0.39, 1.45, Table 3). Further, we did not observe an association between influenza vaccine given alone and infant mortality among term (aHR: 0.77; 95%CI 0.38, 1.53) or preterm (aHR: 0.58; 95%CI 0.34, 1.00) infants. There were no interpretable differences in the estimates after excluding deaths occurring on the day of birth (Supplementary Tables 4 and 5). We were unable to estimate the aHR in preterm infants once the day of birth was excluded from analysis due to small numbers (Supplementary Table 6). Estimates calculated for infants born in NT or Qld generally showed similar patterns but with wider confidence intervals compared to infants born in WA (Supplementary Table 7). By jurisdiction, due to small sample sizes, we were unable to generate stable estimates for infants from the NT for most vaccine combinations. Similarly, we did not have the sample size to calculate effect estimates for cause of death-specific analyses.

**Table 3.** Risk of neonatal and infant mortality associated with prenatal exposure to seasonal inactivated influenza vaccine and/or pertussis vaccine, by prematurity, 1 January 2015 – December 2017.

Age categories	Unexposed infants	Exposed infants by vaccine type			
		Influenza vaccine only	Pertussis vaccine only	Influenza and pertussis vaccine	Influenza or pertussis vaccine
***Among Term infants***					
	**(N=103,540)**	**(N=7,405)**	**(N=82,940)**	**(N=63,517)**	**(N=153, 862)**
***Infant mortality (infants aged 0–365 d)***					
N	103,540	7,405	82,940	63,517	153,862
Deaths, n (no. per 1,000)	174 (1.7)	11 (1.5)	74 (0.9)	58 (0.9)	143 (0.9)
Unweighted HR^a^ (95% CI)	1 [Reference]	0.91 (0.46, 1.77)	0.57 (0.42, 0.73)	0.55 (0.40, 0.75)	0.59 (0.47, 0.73)
Weighted aHR (95% CI)^b^	1 [Reference]	0.77 (0.38, 1.53)	0.62 (0.46, 0.84)	0.65 (0.46, 0.93)	0.76 (0.58, 0.99)
***Early neonatal mortality (infants aged 0–7 d)***					
N	103,540	7,405	82,940	63,517	153,862
Deaths, n (no. per 1,000)	51 (0.5)	<5	24 (0.3)	15 (0.2)	43 (0.3)
Unweighted HR (95% CI)	1 [Reference]	-	0.60 (0.37, 0.99)	0.47 (0.26, 0.85)	0.53 (0.35, 0.81)
Weighted aHR (95% CI)^b^	1 [Reference]	-	0.55 (0.32, 0.95)	0.57 (0.28, 1.17)	0.52 (0.33, 0.83)
***Late neonatal mortality (infants aged 8–28 d)***					
N	103,146	7,330	82,440	62,886	152,656
Deaths, n (no. per 1,000)	24 (0.2)	<5	9 (0.1)	6 (0.1)	16 (0.1)
Unweighted HR (95% CI)	1 [Reference]	-	0.52 (0.24, 1.12)	0.41 (0.17, 1.01)	0.45 (0.23, 0.86)
Weighted aHR (95% CI)^b^	1 [Reference]	-	0.58 (0.25, 1.39)	2.09 (0.35, 12.56)	0.50 (0.24, 1.02)
***Post-neonatal mortality (infants aged 29–365 d)***					
N	102,112	7,159	80,900	60,941	149,000
Deaths, n (no. per 1,000)	99 (1.0)	6 (0.8)	41 (0.5)	37 (0.6)	84 (0.6)
Unweighted HR (95% CI)	1 [Reference]	1.15 (0.50, 2.62)	0.56 (0.38, 0.80)	0.58 (0.38, 0.89)	0.61 (0.45, 0.82)
Weighted aHR (95% CI)^b^	1 [Reference]	0.93 (0.40, 2.20)	0.69 (0.46, 1.02)	0.73 (0.45, 1.16)	0.76 (0.55, 1.06)
***Among Preterm Infants***					
	**(N=11,104)**	**(N=883)**	**(N=5,071)**	**(N=3,519)**	**(N=9,473)**
***Infant mortality (infants aged 0–365 d)***					
N	11,104	883	5,071	3,519	9,473
Deaths, n (no. per 1,000)	362 (32.6)	17 (19.3)	31 (6.1)	14 (4.0)	62 (6.5)
Unweighted HR (95% CI)	1 [Reference]	0.61 (0.36, 1.02)	0.19 (0.13, 0.28)	0.12 (0.07, 0.21)	0.20 (0.15, 0.27)
Weighted aHR (95% CI)^b^	1 [Reference]	0.58 (0.34, 1.00)	0.88 (0.57, 1.36)	0.75 (0.39, 1.45)	0.72 (0.53, 0.98)
***Early neonatal mortality (infants aged 0–7 d)***					
N	11,104	883	5,071	3,519	9,473
Deaths, n (no. per 1,000)	269 (24.2)	9 (10.2)	11 (2.2)	5 (1.4)	25 (2.6)
Unweighted HR (95% CI)	1 [Reference]	0.37 (0.18, 0.79)	0.09 (0.05, 0.17)	0.05 (0.02, 0.13)	0.11 (0.07, 0.16)
Weighted aHR (95% CI)^b^	1 [Reference]	0.38 (0.18, 0.81)	0.55 (0.28, 1.08)	0.31 (0.10, 1.01)	0.49 (0.30, 0.78)
***Late neonatal mortality (infants aged 8–28 d)***					
N	10,769	865	5,026	3,484	9,375
Deaths, n (no. per 1,000)	32 (2.9)	<5	8 (1.6)	<5	14 (1.5)
Unweighted HR (95% CI)	1 [Reference]	-	0.55 (0.25, 1.19)	-	0.51 (0.27, 0.95)
Weighted aHR (95% CI)^b^	1 [Reference]	-	1.70 (0.66, 4.39)	-	1.30 (0.64, 2.64)
***Post-neonatal mortality (infants aged 29–365 d)***					
N	10,591	832	4.907	3,383	9,122
Deaths, n (no. per 1,000)	61 (5.6)	<5	12 (2.4)	7 (2.1)	23 (2.5)
Unweighted HR (95% CI)	1 [Reference]	-	0.49 (0.26, 0.91)	0.44 (0.20, 0.97)	0.51 (0.31, 0.83)
Weighted aHR (95% CI)^b^	1 [Reference]	-	0.96 (0.49, 1.91)	1.45 (0.65, 3.24)	0.94 (0.56, 1.59)

Abbreviations: aHR, adjusted hazard ratio; CI, confidence interval; HR, unadjusted hazard ratio.

^a^Hazard ratios weighted by inverse probability of treatment factoring for maternal covariates including age, Indigenous status, parity, pre-existing medical conditions (asthma, essential hypertension, pre-existing diabetes), pregnancy complications (gestational diabetes, gestational hypertension, pre-eclampsia), smoking status during pregnancy, gestational age at first prenatal care visit, year and season of birth, and socioeconomic advantage as measured by SEIFA quintile^21^; ^b^models were additionally adjusted for the infant’s gestational age.

## Discussion

In this large population-based cohort study with detailed birth, death, perinatal, and immunization data, there was insufficient evidence of greater neonatal or infant mortality associated with influenza or pertussis vaccination during pregnancy. The crude mortality rate of 2.67 deaths/1,000 live births is comparable to published Australian infant mortality rates.^23^ In fact, some of our findings were suggestive of a protective effect against early neonatal death, although these findings should be interpreted with caution and warrant further investigation. These findings provide reassuring evidence that maternal influenza and pertussis vaccination are not associated with increased neonatal or infant mortality and confirm findings from other recent studies. A systematic review summarizing evidence from randomized controlled trials showed no association between the safety of maternal influenza vaccination and infant death.^11^ More recently, a large Danish population cohort study assessing safety outcomes in mothers and infants showed a lower incidence of all-cause neonatal mortality in the vaccinated group.^13^

Our results of the receipt of maternal vaccines and reduced infant mortality could have several possible explanations. While neonatal mortality is determined largely by perinatal events, infections in early life do play a key part in early infant mortality. Severe influenza and pertussis infections and complications occur mostly in very young infants and are more likely to cause severe illness in this age group, resulting in death.^24^ Maternal influenza and pertussis vaccination is important to protect infants at a time they are unable to receive childhood immunizations. Passive immunity via transplacentally maternal antibodies to the newborn and extends the duration of protection until the infant is able to receive their primary immunization series. An analysis by Sandmann et al.^25^ on the cost-effectiveness of the maternal pertussis vaccination program in the UK found that at least 80 infant deaths were saved in the 5-y period 2013–2017 in a cohort of approximately 650,000 infants. A reduced risk of maternal influenza infection during pregnancy via maternal influenza vaccination has also been shown to risk of preterm birth, which is a strong predictor of infant mortality.^26^ Similarly, maternal pertussis vaccination demonstrated high vaccine effectiveness in protecting infants under 3 months old, a period of vulnerability prior to the first childhood pertussis vaccine at 2 months of age.^27^ While it is possible that pertussis and/or influenza are more frequent causes of death than is recorded in mortality data, most infant deaths undergo postmortem examination, including microbiological sampling, and the authors think this explanation is unlikely to be the sole reason for our findings.^28^ We should also acknowledge the possibility of residual confounding in our study. Mothers who get vaccinated during pregnancy have better access to healthcare and attend more antenatal visits, although we did control for the latter.

In addition to direct effects of vaccines against the pathogen of interest, vaccines may also have indirect impacts on infant health. Nonspecific effects of vaccines were first observed with the Bacille-Calmette-Guerin vaccine against Mycobacterium tuberculosis. In addition to effects on tuberculosis, the vaccine has also been observed to provide time-limited partial protection against non-tuberculosis respiratory infections and may reduce mortality.^29^ Nonspecific effects have also been observed with live vaccines such as measles vaccines in low-income settings showing that children who receive MVs early have lower all-cause mortality than can be explained by measles prevention alone.^30^ However, a recent systematic review of randomized controlled trials where high-titer live attenuated measles-containing vaccines were compared to other vaccines or placebo did not support the hypothesis of nonspecific effects of standard-titer measles containing vaccines.^31^ In adjuvanted vaccines using ‘modern’ adjuvants such as Matrix-M and AS0X Adjuvant Systems family, there is some evidence that they induce or modulate nonspecific effects. Matrix-M can trigger inflammasome activation and IL-1ß release and promote antigen cross-presentation.^32^ Similarly, non-clinical evidence that AS01 induces cross-reactive T cell responses (heterologous effect) in a preclinical study.^33^ However, more clinical and epidemiologic confirmation of nonspecific effects of adjuvants is lacking.

Childhood Haemophilus influenza type b and pneumococcal conjugate vaccines^34^ have been shown to have beneficial nonspecific effects, including reductions in community acquired respiratory infections and hospitalizations due to respiratory syncytial virus (RSV), respectively. Pandemic influenza maternal vaccines^35^ have also been shown to reduce gastrointestinal infections. While the role of residual confounding cannot be excluded, a current hypothesis is that currently unexplained immunological factor(s) may induce health benefits beyond disease prevention. It is therefore also possible that maternal influenza and pertussis vaccination also have indirect beneficial impacts on infant health.

We have attempted to minimize the influence of confounding by incorporating a baseline propensity for vaccination and including covariates known to influence health-seeking behavior (vaccination) in pregnant women.^36^ This method has been shown to reduce bias more effectively than conventional multivariable adjustment methods in studies utilizing large administrative databases, such as ours.^22^ We have also conducted several sensitivity analyses to test the robustness of our findings, stratifying by preterm status and excluding the day of birth to account for potentially misclassified stillbirths), but we have also analyzed mortality in the whole cohort for comparison in case this excludes a number of important early neonatal deaths. However, we acknowledge that vaccinated and unvaccinated individuals may differ in ways not fully captured in available data. Alternative study designs, such as comparisons with time periods prior to the introduction of maternal vaccination, could potentially reduce bias introduced by inherent differences in population characteristics. However, such designs may be constrained by other limitations, such as changes in healthcare delivery, or surveillance practices over time.

Strengths of our study include population-based coverage of perinatal, birth, death, and immunization data that capture information on all pregnancies and infants and is representative of the whole population in each jurisdiction. With the exception of the antenatal vaccination register (WAAVD) and jurisdictional immunization registers, the perinatal data collections and births and deaths registers are nationally mandated and provide data to the Australian Institute of Health and Welfare, and the quality is considered to be high.^37^ Furthermore, maternal vaccines in Australia are offered at no cost to all pregnant women, reducing socioeconomic barriers to vaccination. While our study uses older data, Australian trends in infant mortality have not changed drastically over the past 5–10 y,^38^ and our analysis is still relevant in an increasingly crowded maternal vaccine space, and answers frequent questions from community regarding vaccine safety.

However, there were some limitations in our study that should be acknowledged. Data on cause of death were missing for 29% of infants, which limited the sample size for cause-of-death analysis. While we believe outcome misclassification would be uncommon in our study, we cannot exclude the possibility of some exposure misclassification in our analysis.^39^ Maternal vaccination data linked to the WAAVD relied on immunization reports from medical providers. While these records have high specificity, and misclassification of unvaccinated women as vaccinated was uncommon, they are likely to be incomplete, and some vaccinated women may have been misclassified as unvaccinated.^39^ We also did not have information on childhood vaccines and did not adjust for the receipt of childhood influenza and pertussis vaccines. We had low coverage of maternal influenza vaccination and were not able to estimate effect estimates in some instances. We did not control for birthweight in our analysis, which could influence clinical outcomes, particularly at the extremes of the distribution. Our aim in excluding infants <400 g was to reduce heterogeneity due to extreme prematurity or non-viability. Furthermore, only 249 (0.09%) infants <400 g were excluded from the cohort. We acknowledge, however, that infants just above this threshold, as well as those with high birthweights, may still be at risk of suboptimal outcomes. Finally, we did not analyze outcomes by vaccine strain composition.

In conclusion, concerns about the safety of maternal vaccines are often cited by pregnant women as a deterrent to be vaccinated.^36^ Our study provides reassurance that maternal influenza and pertussis vaccines during pregnancy, when received alone or in combination, are not linked with increased infant mortality, and could positively impact on all-cause mortality. However, we note that this analysis does not address non-fatal and long-term adverse outcomes such as developmental disorders, autoimmunity, or other morbidity concerns, which are also of concern to many parents. Mortality is an essential but partial lens for assessing vaccine safety. Nevertheless, these data provide continued support for public maternal vaccination programs. Additional maternal vaccines have been introduced into the vaccination schedule for pregnant women, including COVID-19 vaccines for high-risk pregnant women and RSV vaccines for all pregnant women.^5^ As a result, safety studies on the receipt of multiple maternal vaccines during pregnancy are becoming increasingly important to provide reassurance to and support vaccine decision-making by pregnant women and their healthcare professionals and should include a range of outcomes in their assessment.

## Supplementary Material

Supplemental Material

## Data Availability

The data included in this study are not publicly available and are only made available to researchers through an application process. However, analytic code used in this study can be made available upon request.
