# Supplementary material for: Neonatal and infant mortality after maternal influenza and pertussis vaccination: Probabilistically linked cohort study
Source: Hum Vaccin Immunother. 2025 Nov 20;21(1):2587307. doi: 10.1080/21645515.2025.2587307 (PMC12645885; doi:10.1080/21645515.2025.2587307)
Supplement: Supplemental Material [file KHVI_A_2587307_SM1813.docx]

**Neonatal and infant mortality after maternal influenza and pertussis vaccination: probabilistically linked cohort study**

Mohinder Sarna^1,2^, Christopher C Blyth^2,3,4^, Hannah C Moore^1,2^, Gavin Pereira^2,5^, Lisa McHugh^6^, Michael Binks^7,8^, Karin Lust^9,10^, Paul Van Buynder^11^, Damien Foo^1,12,13^, Ross Andrews^14^, Annette K Regan^1,2,15,16^

**Supplementary section**

**Supplementary Figure 1:** Balance of maternal covariates before and after inverse probability treatment weighting by type of vaccine. a) influenza vaccine only during pregnancy; b) pertussis vaccine only during pregnancy; c) influenza and pertussis vaccine during pregnancy

**Supplementary Table 1:** Jurisdictional data sources used in this analysis

**Supplementary Table 2:** ICD-10 codes used to classify specific cause of death

**Supplementary Table 3: Influenza vaccine composition recommended during the study period for Australian Southern Hemisphere by the World Health Organization**

**Supplementary Table 4:** Risk of neonatal and infant mortality associated with prenatal exposure to maternal seasonal inactivated influenza vaccine and/or pertussis vaccine after excluding deaths occurring on the first day after birth, 1 January 2015 – December 2017

**Supplementary Table 5:** Risk of neonatal and infant mortality associated with prenatal exposure to maternal seasonal inactivated influenza vaccine and/or pertussis vaccine among term infants after excluding deaths occurring on the first day after birth, 1 January 2015 – December 2017

**Supplementary Table 6:** Risk of neonatal or infant mortality associated with prenatal exposure to maternal seasonal inactivated influenza vaccine and/or pertussis vaccine among preterm infants after excluding deaths occurring on the first day after birth, 1 January 2015 – December 2017

**Supplementary Table 7:** Risk of infant mortality associated with prenatal exposure to maternal influenza and pertussis vaccines among all infants in the first year of life by Australian jurisdiction and age sub-group, 1 January 2015 – 31 December 2017.

**Supplementary Figure 1: Balance of maternal covariates before and after inverse probability treatment weighting by type of vaccine. a) influenza vaccine only during pregnancy; b) pertussis vaccine only during pregnancy; c) influenza and pertussis vaccine during pregnancy**


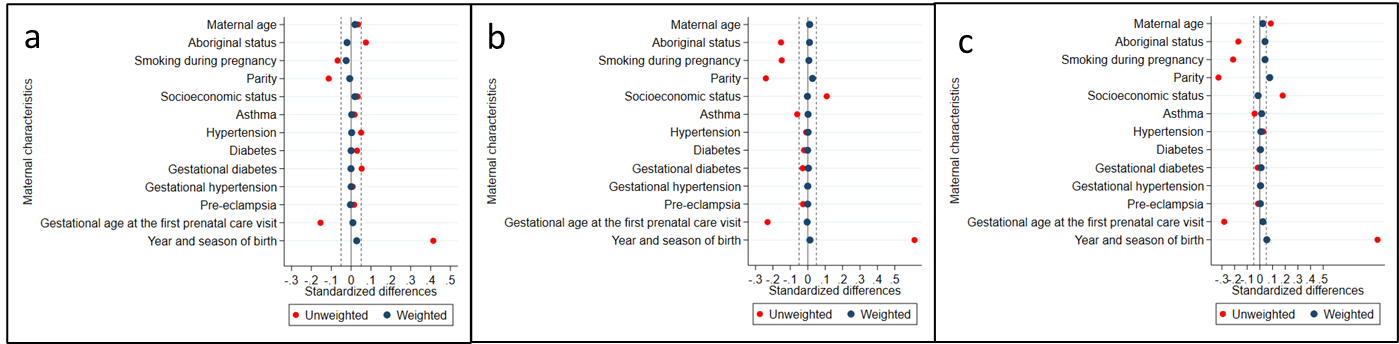


**Supplementary Table 1: Jurisdictional data sources used in this analysis**

| **Data sources** | **Queensland** | **Northern Territory** | **Western Australia** |
| --- | --- | --- | --- |
| Perinatal data collection | Perinatal Data Collection | Perinatal Trends | Midwives Notification System |
| Antenatal vaccination | Vaccination Information and Vaccination Administration System | NT Immunisation Register | Midwives Notification system  WA Antenatal Vaccination Database |
| Birth registrations | Registrar General Births | Birth Registry | Birth Registrations |
| Death registrations | Registrar General Deaths | Deaths Registry | Death Registry |

**Supplementary Table 2: ICD-10 codes used to classify specific causes of death (n=528)**

| **Cause of Death categories** | | **ICD-10 codes** | **Number, (%)** |
| --- | --- | --- | --- |
| Infection/sepsis | |  | 22 (4.2) |
|  | Certain infectious and parasitic diseases | A00-B99 |  |
|  | Acute upper respiratory infections | J00-J06 |  |
|  | Influenza and pneumonia | J09-J18 |  |
|  | Other lower respiratory tract infections | J20-22 |  |
|  | Pertussis | A37.9 |  |
|  | Sepsis | P36* |  |
| Neoplasms | | C00-D48 | 10 (1.9) |
| Disorders of the nervous system | | G00-G99 | 15 (2.8) |
| Disorders of the circulatory system | | I00-I99 | 12 (2.3) |
| Conditions originating in perinatal period | | P00-P96 | 227 (43.0) |
|  | Preterm birth | P07.2*, P07.3* | 32 (5.7) |
| Congenital abnormalities | | Q00-Q99 | 158 (40.5) |
| Sudden infant death syndrome | | R95 | 21 (3.8) |
| Ill-defined and unknown cause of mortality | | R99 | 31 (5.5) |
| Accidents, injuries and trauma | | V00-Y33 | 24 (4.3) |
| Other causes of death^#^ | | - | <10 |

*indicates all codes with this prefix; # individual causes of death not presented as small number suppression was employed**Supplementary Table 3: Influenza vaccine composition recommended during the study period for Australian Southern Hemisphere by the World Health Organization**

| **Year** | **Influenza^1^** |
| --- | --- |
| **2015** | **TIV:** A/California/7/2009 (H1N1)pdm09; A/Switzerland/9715293/2013 (H3N2); B/Phuket/3073/2013 (B/Yamagata).  **+QIV adds:** B/Brisbane/60/2008 (B/Victoria)[1] |
| **2016** | **TIV:** A/California/7/2009 (H1N1)pdm09; A/Hong Kong/4801/2014 (H3N2); B/Brisbane/60/2008 (B/Victoria).  **+QIV adds:** B/Phuket/3073/2013 (B/Yamagata)[2] |
| **2017** | **TIV:** A/Michigan/45/2015 (H1N1)pdm09; A/Hong Kong/4801/2014 (H3N2); B/Brisbane/60/2008 (B/Victoria).  **+ QIV adds:** B/Phuket/3073/2013 (B/Yamagata)[3] |

^1^Strains recommended for Australia’s Southern Hemisphere influenza vaccines by the World Health Organization and advised by Australian Influenza Vaccine Committee via the Therapeutic Goods Administration. Trivalent components are listed first; where quadrivalent vaccine was used, the second B lineage added that year is listed after ‘+QIV adds’

Abbreviations: QIV: quadrivalent influenza vaccine; TIV: trivalent influenza vaccine

**References**

1. World Health Organization. Recommended composition of influenza virus vaccines for use in the 2015 southern hemisphere influenza season. Available at: <https://www.who.int/docs/default-source/influenza/who-influenza-recommendations/vcm-southern-hemisphere-recommendation-2015/201409_recommendation.pdf>.

2. World Health Organization. Recommended composition of influenza virus vaccines for use in the 2016 southern hemisphere influenza season. Available at: <https://cdn.who.int/media/docs/default-source/influenza/who-influenza-recommendations/vcm-southern-hemisphere-recommendation-2016/201509_recommendation.pdf>.

3. World Health Organization. Recommended composition of influenza virus vaccines for use in the 2017 southern hemisphere influenza season. Available at: <https://cdn.who.int/media/docs/default-source/influenza/who-influenza-recommendations/vcm-southern-hemisphere-recommendation-2017/201609_recommendation.pdf>.

**Supplementary Table 4: Risk of neonatal and infant mortality associated with prenatal exposure to maternal seasonal inactivated influenza vaccine and/or pertussis vaccine after excluding deaths occurring on the first day after birth, 1 January 2015 – December 2017**

| **Age categories** | **Unexposed infants**  **(N=114,424)** | **Exposed infants by vaccine type** | | | |
| --- | --- | --- | --- | --- | --- |
|  |  | **Influenza vaccine only**  **(N=8,283)** | **Pertussis vaccine only**  **(N=88,000)** | **Influenza and pertussis vaccine (N=67,031)** | **Influenza or pertussis vaccine (N=163,314)** |
| ***Infant mortality (infants aged 1-365 days)*** | | | | | |
| N | 114,424 | 8,283 | 88,000 | 67,031 | 163,314 |
| Deaths, n (no. per 1,000) | 316 (2.8) | 23 (2.8) | 94 (1.1) | 67 (1.0) | 184 (1.1) |
| Unweighted HR^1^ (95% CI) | 1 [Reference] | 1.08 (0.69, 1.70) | 0.40 (0.32, 0.51) | 0.36 (0.27, 0.48) | 0.42 (0.35, 0.51) |
| Weighted aHR (95% CI)^2^ | 1 [Reference] | 0.85 (0.53, 1.36) | 0.72 (0.55, 0.94) | 0.70 (0.51, 0.97) | 0.72 (0.58, 0.90) |
| ***Early neonatal mortality (infants aged 1-7 days)*** | | | | | |
| N | 114,424 | 8,283 | 88,000 | 67,031 | 163,314 |
| Deaths, n (no. per 1,000) | 100 (0.9) | 8 (1.0) | 24 (0.3) | 15 (0.2) | 47 (0.3) |
| Unweighted HR (95% CI) | 1 [Reference] | 0.80 (0.33, 1.97) | 0.31 (0.19, 0.48) | 0.23 (0.13, 0.41) | 0.29 (0.20, 0.42) |
| Weighted aHR (95% CI)^2^ | 1 [Reference] | 0.66 (0.27, 1.64) | 0.60 (0.35, 1.02) | 0.53 (0.27, 1.03) | 0.54 (0.35, 0.85) |
| ***Late neonatal mortality (infants aged 8-28 days)*** | | | | | |
| N | 113,915 | 8,195 | 87,466 | 66,370 | 162,031 |
| Deaths, n (no. per 1,000) | 56 (0.5) | 5 (0.6) | 17 (0.2) | 8 (0.1) | 30 (0.2) |
| Unweighted HR (95% CI) | 1 [Reference] | 1.45 (0.58, 3.63) | 0.41 (0.24, 0.71) | 0.22 (0.10, 0.49) | 0.38 (0.24, 0.60) |
| Weighted aHR (95% CI)^2^ | 1 [Reference] | 1.17 (0.45, 3.07) | 0.88 (0.44, 1.74) | 0.36 (0.16, 0.84) | 0.75 (0.43, 1.32) |
| ***Post-neonatal mortality (infants aged 29-365 days)*** | | | | | |
| N | 112,703 | 7,991 | 85,807 | 64,324 | 158,122 |
| Deaths, n (no. per 1,000) | 160 (1.4) | 7 (0.9) | 50 (0.6) | 37 (0.6) | 100 (0.6) |
| Unweighted HR (95% CI) | 1 [Reference] | 1.16 (0.61, 2.20) | 0.47 (0.35, 0.65) | 0.47 (0.33, 0.68) | 0.52 (0.40, 0.67) |
| Weighted aHR (95% CI)^2^ | 1 [Reference] | 0.87 (0.44, 1.70) | 0.76 (0.53, 1.07) | 0.83 (0.54, 1.27) | 0.82 (0.61, 1.09) |
| Abbreviations: aHR, adjusted hazard ratio; CI, confidence interval; HR, unadjusted hazard ratio.  ^1^Hazard ratios weighted by inverse-probability of treatment factoring for maternal covariates including age, Indigenous status, parity, pre-existing medical conditions (asthma, essential hypertension, pre-existing diabetes), pregnancy complications (gestational diabetes, gestational hypertension, pre-eclampsia), smoking status during pregnancy, gestational age at first prenatal care visit, year and season of birth, and socioeconomic advantage as measured by SEIFA quintile^24^; ^2^models were additionally adjusted for the infant’s gestational age | | | | | |

**Supplementary Table 5: Risk of neonatal and infant mortality associated with prenatal exposure to maternal seasonal inactivated influenza vaccine and/or pertussis vaccine among term infants after excluding deaths occurring on the first day after birth, 1 January 2015 – December 2017**

| **Age categories** | **Unexposed infants**  **(N=103,521)** | **Exposed infants by vaccine type** | | | |
| --- | --- | --- | --- | --- | --- |
|  |  | **Influenza vaccine only**  **(N=7,405)** | **Pertussis vaccine only**  **(N=82,932)** | **Influenza and pertussis vaccine**  **(N=63,513)** | **Influenza or pertussis vaccine**  **(N=153,850)** |
| ***Infant mortality (infants aged 1-365 days)*** | | | | | |
| N | 103,521 | 7,405 | 82,932 | 63,513 | 153,850 |
| Deaths, n (no. per 1,000) | 155 (1.5) | 11 (1.5) | 66 (0.8) | 54 (0.9) | 131 (0.9) |
| Unweighted HR^1^ (95% CI) | 1 [Reference] | 1.03 (0.52, 2.01) | 0.56 (0.42, 0.75) | 0.58 (0.42, 0.80) | 0.59 (0.47, 0.75) |
| Weighted aHR (95% CI)^2^ | 1 [Reference] | 0.86 (0.43, 1.72) | 0.62 (0.45, 0.85) | 0.66 (0.46, 0.95) | 0.67 (0.52, 0.88) |
| ***Early neonatal mortality (infants aged 1-7 days)*** | | | | | |
| N | 103,521 | 7,405 | 82,932 | 63,513 | 153,850 |
| Deaths, n (no. per 1,000) | 32 (0.3) | <5 | 16 (0.2) | 11 (0.2) | 31 (0.2) |
| Unweighted HR (95% CI) | 1 [Reference] | - | 0.63 (0.34, 1.17) | 0.55 (0.27, 1.13) | 0.61 (0.36, 1.02) |
| Weighted aHR (95% CI)^2^ | 1 [Reference] | - | 0.52 (0.27, 1.02) | 0.57 (0.26, 1.23) | 0.56 (0.31, 0.99) |
| ***Late neonatal mortality (infants aged 8-28 days)*** | | | | | |
| N | 103,146 | 7,330 | 82,440 | 62,886 | 152,656 |
| Deaths, n (no. per 1,000) | 24 (0.2) | <5 | 9 (0.1) | 6 (0.1) | 16 (0.1) |
| Unweighted HR (95% CI) | 1 [Reference] | - | 0.52 (0.24, 1.12) | 0.36 (0.14, 0.96) | 0.45 (0.23, 0.86) |
| Weighted aHR (95% CI)^2^ | 1 [Reference] | - | 0.58 (0.25, 1.39) | 0.32 (0.12, 0.87) | 0.50 (0.24, 1.02) |
| ***Post-neonatal mortality (infants aged 29-365 days)*** | | | | | |
| N | 102,112 | 7,159 | 80,900 | 60,941 | 149,000 |
| Deaths, n (no. per 1,000) | 99 (1.0) | <5 | 39 (0.5) | 31 (0.5) | 78 (0.5) |
| Unweighted HR (95% CI) | 1 [Reference] | - | 0.56 (0.38, 0.80) | 0.58 (0.38, 0.89) | 0.61 (0.45, 0.82) |
| Weighted aHR (95% CI)^2^ | 1 [Reference] | - | 0.69 (0.46, 1.02) | 0.73 (0.45, 1.16) | 0.76 (0.55, 1.06) |
| Abbreviations: aHR, adjusted hazard ratio; CI, confidence interval; HR, unadjusted hazard ratio.  ^1^Hazard ratios weighted by inverse-probability of treatment factoring for maternal covariates including age, Indigenous status, parity, pre-existing medical conditions (asthma, essential hypertension, pre-existing diabetes), pregnancy complications (gestational diabetes, gestational hypertension, pre-eclampsia), smoking status during pregnancy, gestational age at first prenatal care visit, year and season of birth, and socioeconomic advantage as measured by SEIFA quintile^24^; ^2^models were additionally adjusted for the infant’s gestational age | | | | | |

**Supplementary Table 6: Risk of neonatal or infant mortality associated with prenatal exposure to maternal seasonal inactivated influenza vaccine and/or pertussis vaccine among preterm infants after excluding deaths occurring on the first day after birth, 1 January 2015 – December 2017**

| **Age categories** | **Unexposed infants**  **(N=10,903)** | **Exposed infants by vaccine type** | | | |
| --- | --- | --- | --- | --- | --- |
|  |  | **Influenza vaccine only**  **(N=878)** | **Pertussis vaccine only**  **(N=5,068)** | **Influenza and pertussis vaccine**  **(N=3,518)** | **Influenza or pertussis vaccine (N=9,464)** |
| ***Infant mortality (infants aged 1-365 days)*** | | | | | |
| N | 10,903 | 878 | 5,068 | 3,518 | 9,464 |
| Deaths, n (no. per 1,000) | 161 (15.0) | 12 (13.9) | 28 (5.6) | 13 (3.7) | 53 (5.6) |
| Unweighted HR (95% CI) | 1 [Reference] | 1.02 (0.56, 1.88) | 0.39 (0.26, 0.59) | 0.25 (0.14, 0.46) | 0.40 (0.29, 0.54) |
| Weighted aHR (95% CI)^2^ | 1 [Reference] | 0.91 (0.46, 1.79) | 1.03 (0.65, 1.64) | 0.92 (0.47, 0.73) | 0.90 (0.63, 1.29) |
| ***Early neonatal mortality (infants aged 1-7 days)*** | | | | | |
| N | 10,903 | 878 | 5,068 | 3,518 | 9,464 |
| Deaths, n (no. per 1,000) | 68 (6.3) | <5 | 8 (1.6) | <5 | 16 (1.7) |
| Unweighted HR (95% CI) | 1 [Reference] | - | 0.26 (0.12, 0.53) | - | 0.25 (0.15, 0.44) |
| Weighted aHR (95% CI)^2^ | 1 [Reference] | - | 0.76 (0.34, 1.71) | - | 0.66 (0.34, 1.27) |
| ***Late neonatal mortality (infants aged 8-28 days)*** | | | | | |
| N | 10,769 | 865 | 5,026 | 3,484 | 9,375 |
| Deaths, n (no. per 1,000) | 32 (2.9) | <5 | 8 (1.6) | <5 | 14 (1.5) |
| Unweighted HR (95% CI) | 1 [Reference] | - | 0.55 (0.25, 1.19) | - | 0.50 (0.27, 0.95) |
| Weighted aHR (95% CI)^2^ | 1 [Reference] | - | 1.70 (0.66, 4.41) | - | 1.30 (0.64, 2.64) |
| ***Post-neonatal mortality (infants aged 29-365 days)*** | | | | | |
| N | 10,591 | 832 | 4,907 | 3,383 | 9,122 |
| Deaths, n (no. per 1,000) | 61 (5.6) | <5 | 11 (2.2) | 6 (1.8) | 22 (2.4) |
| Unweighted HR (95% CI) | 1 [Reference] | - | 0.49 (0.26, 0.91) | 0.44 (0.20, 0.97) | 0.52 (0.32, 0.84) |
| Weighted aHR (95% CI)^2^ | 1 [Reference] | - | 0.96 (0.49, 1.91) | 1.42 (0.64, 3.19) | 0.96 (0.57, 1.62) |
| Abbreviations: aHR, adjusted hazard ratio; CI, confidence interval; HR, unadjusted hazard ratio.  ^1^Hazard ratios weighted by inverse-probability of treatment factoring for maternal covariates including age, Indigenous status, parity, pre-existing medical conditions (asthma, essential hypertension, pre-existing diabetes), pregnancy complications (gestational diabetes, gestational hypertension, pre-eclampsia), smoking status during pregnancy, gestational age at first prenatal care visit, year and season of birth, and socioeconomic advantage as measured by SEIFA quintile^24^; ^2^models were additionally adjusted for the infant’s gestational age | | | | | |

**Supplementary Table 7: Risk of infant mortality associated with prenatal exposure to maternal seasonal influenza and pertussis vaccines among all infants in the first year of life by Australian jurisdiction and age sub-group, 1 January 2015 – 31 December 2017**

| **Jurisdiction** | **Unexposed infants** | **Exposed infants by vaccine type** | | | |  |
| --- | --- | --- | --- | --- | --- | --- |
|  |  | **Influenza vaccine only** | **Pertussis vaccine only** | **Influenza and pertussis vaccine** | **Influenza or pertussis vaccine** |  |
| ***Queensland*** |  |  |  |  |  |  |
| N | 60,162 | 3,723 | 66,714 | 44,827 | 115,264 |  |
| Deaths, n (no. per 1,000) | 361 (6.0) | 20 (5.4) | 84 (1.3) | 41 (0.9) | 145 (1.3) |  |
| Unweighted HR^1^ (95% CI) | 1 [Reference] | 0.94 (0.58, 1.52) | 0.21 (0.17, 0.27) | 0.16 (0.11, 0.22) | 0.23 (0.19, 0.27) |  |
| Weighted aHR (95% CI)^2^ | 1 [Reference] | 0.82 (0.50, 1.35) | 0.65 (0.48, 0.88) | 0.57 (0.38, 0.88) | 0.66 (0.51, 0.86) |  |
| ***Northern Territory*** |  |  |  |  |  |  |
| N | 6,677 | 744 | 1,708 | 735 | 3,187 |  |
| Deaths, n (no. per 1,000) | 38 (5.7) | <5 | <5 | <5 | 5 (1.6) |  |
| Unweighted HR (95% CI) | 1 [Reference] | - | - | - | 0.30 (0.12, 0.76) |  |
| Weighted aHR (95% CI)^2^ | 1 [Reference] | - | - | - | 0.32 (0.11, 0.94) |  |
| ***Western Australia*** |  |  |  |  |  |  |
| N | 47,805 | 3,821 | 19,589 | 21,474 | 44,884 |  |
| Deaths, n (no. per 1,000) | 137 (2.9) | 6 (1.6) | 19 (1.0) | 30 (1.4) | 55 (1.2) |  |
| Unweighted HR (95% CI) | 1 [Reference] | 0.54 (0.22, 1.33) | 0.34 (0.21, 0.56) | 0.45 (0.29, 0.69) | 0.42 (0.30, 0.58) |  |
| Weighted aHR (95% CI)^2^ | 1 [Reference] | 0.45 (0.18, 1.09) | 0.61 (0.36, 1.05) | 0.96 (0.58, 1.60) | 0.77 (0.53, 1.10) |  |
| Abbreviations: CI, confidence interval; HR, unadjusted hazard ratio; aHR, adjusted hazard ratio.  ^a^Hazard ratios weighted by inverse-probability of treatment factoring for maternal covariates including age, Indigenous status, parity, pre-existing medical conditions (asthma, essential hypertension, pre-existing diabetes), pregnancy complications (gestational diabetes, gestational hypertension, pre-eclampsia), smoking status during pregnancy, gestational age at first prenatal care visit, year and season of birth, and socioeconomic advantage as measured by SEIFA quintile^24^; ^2^models were additionally adjusted for the infant’s gestational age | | | | | | |
